# Assessing the impact of biomedical research in academic institutions of disparate sizes

**DOI:** 10.1186/1471-2288-9-33

**Published:** 2009-05-29

**Authors:** Vana Sypsa, Angelos Hatzakis

**Affiliations:** 1 Department of Hygiene and Epidemiology, Athens University Medical School, Athens, Greece

## Abstract

**Background:**

The evaluation of academic research performance is nowadays a priority issue. Bibliometric indicators such as the number of publications, total citation counts and h-index are an indispensable tool in this task but their inherent association with the size of the research output may result in rewarding high production when evaluating institutions of disparate sizes. The aim of this study is to propose an indicator that may facilitate the comparison of institutions of disparate sizes.

**Methods:**

The Modified Impact Index (MII) was defined as the ratio of the observed h-index (*h*) of an institution over the h-index anticipated for that institution on average, given the number of publications (*N*) it produces i.e.  (*α *and *β *denote the intercept and the slope, respectively, of the line describing the dependence of the h-index on the number of publications in log_10 _scale). MII values higher than 1 indicate that an institution performs better than the average, in terms of its *h*-index. Data on scientific papers published during 2002–2006 and within 36 medical fields for 219 Academic Medical Institutions from 16 European countries were used to estimate *α *and *β *and to calculate the MII of their total and field-specific production.

**Results:**

From our biomedical research data, the slope *β *governing the dependence of h-index on the number of publications in biomedical research was found to be similar to that estimated in other disciplines (≈0.4). The MII was positively associated with the average number of citations/publication (r = 0.653, p < 0.001), the h-index (r = 0.213, p = 0.002), the number of publications with ≥ 100 citations (r = 0.211, p = 0.004) but not with the number of publications (r = -0.020, p = 0.765). It was the most highly associated indicator with the share of country-specific government budget appropriations or outlays for research and development as % of GDP in 2004 (r = 0.229) followed by the average number of citations/publication (r = 0.153) whereas the corresponding correlation coefficient for the h-index was close to 0 (r = 0.029). MII was calculated for first 10 top-ranked European universities in life sciences and biomedicine, as provided by Times Higher Education ranking system, and their total and field-specific performance was compared.

**Conclusion:**

The MII should complement the use of h-index when comparing the research output of institutions of disparate sizes. It has a conceptual interpretation and, with the data provided here, can be computed for the total research output as well as for field-specific publication sets of institutions in biomedicine.

## Background

Bibliometric indices are an indispensable tool in evaluating the research output of individuals and institutions. Recently, novel indicators have been proposed with the aim to overcome deficiencies of the "traditional" bibliometric indices (e.g. number of publications, total citation count, average number of citations per publication) and to combine more efficiently information on both the quantity and the quality of the research output [1-4]. H-index is the most known example of such an indicator [1] and is now routinely provided by Thomson Scientific Web of Science and other bibliometric databases. This indicator is defined as the number *h *of papers of an individual or an institution with number of citations higher or equal to *h*. As a result, it combines information on both the number of papers and the number of citations. However, due to its inherent association with the size of the research output it may result in rewarding institutions with high production [2]. Thus, when comparing institutions, a proper calibration of the h-index for the size of the output may provide additional information.

Recenlty, it has been shown that when evaluating sets of publications ranging from several hundreds to 10^5 ^papers, the dependence of the h-index on the size of the set is characterised by a "universal" growth rate [2]. This was shown for interdisciplinary, mechanics and materials science data [2] as well as for nonbiomedical research data [5]. Thus, the h-index can be decomposed into the product of a factor depending on the population size and of an impact index. This impact index can be used to compare the research output of institutions of disparate number of publications. However, as most bibliometric indicators, the impact index of an institution is not informative on its own, unless it is compared to the corresponding indices of other institutions. Furthermore, Molinari and Molinari [2] have provided parameter estimates to calculate this index only for a large number of papers and therefore, it cannot be extended to assess the impact in e.g. specific fields where the sets of publications range on a much lower scale.

In the present study we aim to extend the interpretation of the h-index by proposing a size-corrected, h-index based indicator (Modified Impact Index – MII). The concept of this index is to assess whether the h-index of an institution deviates from the average h-index, as estimated for a particular number of publications. MII shares all the merits of the impact index. Additionally, we will show that it has a more informative numerical interpretation and, with the data that we will provide in the following sections, it may be used also in the case of smaller publication sets. We will illustrate the use of this index in biomedical research and explore its application within specific biomedical disciplines.

## Methods

The Academic Medical Institutions located in 16 European countries (Austria, Belgium, Denmark, Finland, France, Germany, Greece, Ireland, Italy, Netherlands, Norway, Portugal, Spain, Sweden, Switzerland, United Kingdom) were identified from the database of medical schools provided by the Institute for International Medical Education [6]. Once the final list of 219 institutions was compiled, all publications affiliated to the corresponding universities (excluding meeting abstracts) and classified into any of the 36 pre-specified medical subjects (Table 1) were identified using Thomson Scientific Web of Science (WoS). The number of papers published during 2002–2006 and the corresponding h-index have been recorded for each institution. Two databases have been constructed; one with data on all publications within the 36 medical fields and a second with data on publications from each medical field separately. The intercept *α *and slope *β *of the line describing the dependence of h-index on the number of publications (log_10 _scale) were obtained through least-squares estimation.

**Table 1:** List of 36 medical subjects included in the evaluation along with the estimated *α* s and *β *s (as obtained from data on publications of 219 European Academic Medical Institutions within 2002–2006) for the calculation of the modified impact index ( where *h*: h-index, *N*: number of publications)

	Subject	Intercept *α*	Slope *β*
1	Allergy	-0.033	0.668

2	Anatomy & Morphology	-0.058	0.623

3	Anesthesiology	-0.016	0.554

4	Cardiac & Cardiovascular Systems	-0.004	0.600

5	Chemistry, Medicinal	0.067	0.563

6	Clinical Neurology	0.027	0.545

7	Critical Care Medicine	0.053	0.594

8	Dermatology	-0.031	0.560

9	Emergency Medicine	-0.016	0.498

10	Endocrinology & Metabolism	0.098	0.560

11	Gastroenterology & Hepatology	0.028	0.592

12	Geriatrics & Gerontology	0.049	0.558

13	Health Care Sciences & Services	-0.022	0.538

14	Hematology	0.046	0.614

15	Immunology	0.162	0.528

16	Infectious Diseases	0.075	0.566

17	Medicine, General & Internal	-0.124	0.644

18	Medicine, Research & Experimental	-0.008	0.621

19	Obstetrics & Gynecology	0.040	0.521

20	Oncology	0.205	0.500

21	Ophthalmology	-0.055	0.581

22	Orthopedics	-0.053	0.555

23	Otorhinolaryncology	0.004	0.488

24	Pathology	-0.042	0.621

25	Pediatrics	-0.027	0.528

26	Peripheral Vascular Disease	0.022	0.616

27	Physiology	0.062	0.565

28	Psychiatry	-0.012	0.566

29	Public, Environmental & Occupational Health	0.020	0.535

30	Radiology, Nuclear Medicine & Medical Imaging	0.001	0.560

31	Respiratory System	-0.025	0.607

32	Rheumatology	-0.006	0.638

33	Surgery	0.070	0.490

34	Transplantation	0.006	0.572

35	Tropical Medicine	0.003	0.595

36	Urology & Nephrology	-0.012	0.594

The impact index of each institution was calculated as  where *h*: h-index and *N*: number of publications. As Molinari and Molinari have shown in their paper [2], the slope *β *of 0.4 estimated when accumulating data on h-index over time is similar to the slope of the regression line obtained from cross-sectional data (e.g. in their paper: h-index per country as calculated in 2006 vs. the corresponding number of publications). Thus, we used the latter approach and estimated the impact index of papers published within 2002–2006 using the slope *β *obtained from our data on 219 institutions.

To illustrate our findings, we used the rankings provided by Times Higher Education to select top-ranked European universities in life sciences and biomedicine [7].

## Results

### Modified Impact Index (MII) in biomedical research

**Figure 1:**
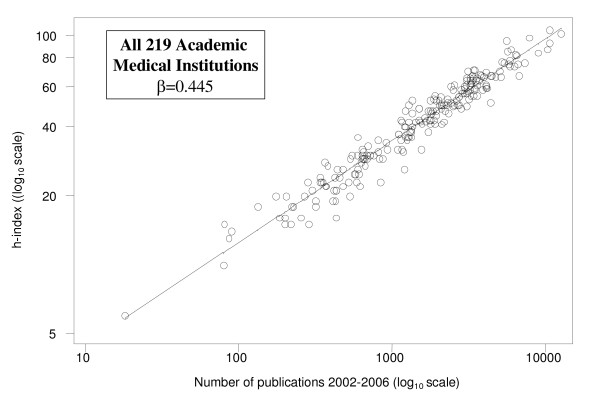
**Log-log plot of h-index versus the total number of results found in 219 Medical schools from 16 European countries**. The solid line indicates the fitted regression line and *β *indicates its slope.

**Figure 2:**
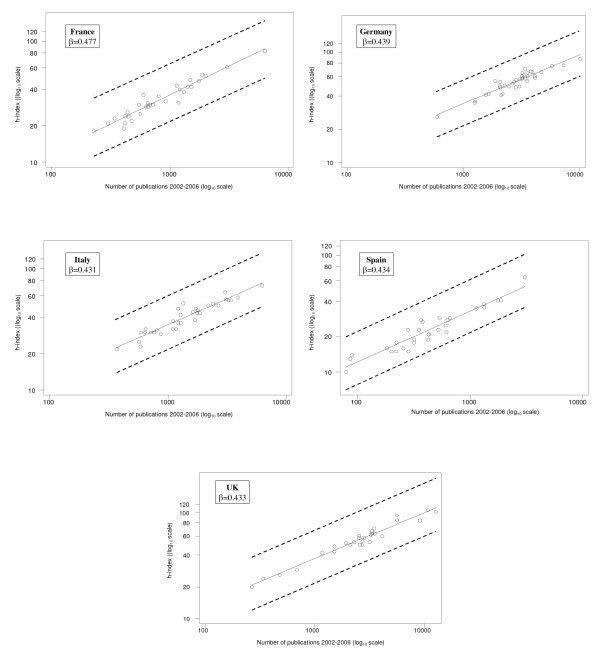
**Log-log plot of h-index versus the total number of results by country (including countries with more than 10 Academic Medical Institutions)**. The solid line indicates the fitted regression line and *β *indicates its slope. Overlaid are lines of slope equal to 0.445.

When the h-index of each institution was plotted against the corresponding number of papers from 36 medical fields on a log-log plot, the resulting points were fitted by a regression line (Figure 1):(1)

where *h*_*i *_and *N*_*i *_the h-index and the number of publications of the i^th ^institution, respectively, *α *and *β *the intercept and the slope of the regression line and *ε*_*i *_the *i*^th ^residual. The estimated *α *and *β *were 0.207 and 0.445, respectively. The parameter *β *= 0.445 governing the dependence of h-index on the number of publications in biomedical research was found to be similar to that estimated in other disciplines (≈0.4). The number of publications ranged from 10^2 ^to 10^4 ^papers, with the exception of one institution with very low number of publications. The exclusion of this institution did not alter the estimated slope. Our estimate for *β *in biomedical sciences was consistent among different countries (Figure 2).

The fitted regression line of equation (1) provides the average h-index for a particular number of publications. Thus, points above the regression line correspond to institutions with h-index higher than the average. Similarly, points below the regression line correspond to institutions with h-index lower than the average. The difference log_10 _*h*_*i *_- (*α *+ *β *log_10 _*N*_*i*_) between the observed log_10 _*h*_*i *_(denoted as circles in the Figure 1 and 2) and the corresponding fitted value *α *+ *β *log_10 _*N*_*i *_(superimposed regression line) expresses the deviation *ε*_*i *_of the observed h-index of the *i*^th ^institution from the average estimate for the number of publications it produces. In the original scale, this difference is transformed into the ratio . This ratio expresses how many times the observed *h*-index is higher than that estimated by the regression model based on the number of publications. Thus, a value higher than 1 indicates that the particular institution performs better in terms of *h*-index than it would be expected for the number of publications it produces. Similarly, a value lower than 1 indicates that the particular institution performs worse in terms of *h*-index than it would be expected for the number of publications it produces. The ratio  was found to be equivalent to the impact index  proposed by Molinari and Molinari [2] multiplied by the constant  and was therefore named Modified Impact Index (MII). The variance of the MII can be computed as follows. In log_10 _scale:

In the original scale  and thus it follows the lognormal distribution. From standard theory, . Based on the data collected from 219 European Medical Institutions, *Var*(MII) was estimated to be equal to 0.013475.

We explored the validity of the MII by examining its association with other indices. The MII was positively associated with the average number of citations/publication (Spearman's r = 0.653, p < 0.001), the h-index (r = 0.213, p = 0.002), the number of publications with ≥ 100 citations (r = 0.211, p = 0.004) but not with the number of publications (r = -0.020, p = 0.765). We further examined whether the country-specific modified impact indices (calculated as the median of the MIIs of the institutions for each country) correlated with the share of government budget appropriations or outlays for research and development (GBAORD) as % of GDP in 2004 (GBAORD are a way of measuring government support to R&D activities) [8]. The MII was the most highly associated indicator (r = 0.229) followed by the average number of citations/publication (r = 0.153) whereas the corresponding correlation coefficient for the h-index was close to 0 (r = 0.029).

### MII in specific medical subfields

When evaluating the MII or the impact index of an institution within a specific medical field, the value of the slope *β *may not be necessarily equal to 0.445 as the range of the evaluated sets of publications lies on a much lower scale. The evaluated sets of publications per field for the years 2002–2006 ranged from less than 10 papers to several hundreds (on average up to 500 papers) as opposed to the range of 10^2^–10^4 ^papers for all 36 subjects. Some fields were characterized by a small range in the number of publications (e.g. Anatomy with range over 219 institutions up to 76 papers) and others reached thousands (e.g. Medicine General and Internal with range up to 1063 papers).

We used the database with the number of publications and corresponding h-indices per subfield. Plots similar to Figure 1 were constructed for each one of the 36 medical fields and the parameters *α *and *β *were estimated (Table 1). These parameters can be used to estimate the field-specific MII of an institution or a department. The field-specific slopes had a mean (SD) of 0.571 (0.045) and ranged from 0.488 (subfield: Otorhinolaryncology) to 0.668 (subfield: Allergy). There was a slight negative association between the slopes and the number of publications per field (i.e. higher slopes in sub fields with few publications), which was not statistically significant (r = -0.126, p = 0.465).

### MII for selected top-ranked universities

To illustrate our findings, we compared the first 10 top-ranked European universities in life sciences and biomedicine, as provided by Times Higher Education [7]. In Table 2, the number of publications, h-index, impact index proposed by Molinari [2] and MII are presented for all 36 medical fields (publication years: 2002–2006). All universities had a MII higher than 1 (range: 1.027–1.403) i.e. their performance based on the h-index was higher than or around that expected based on the number of papers they produced. In terms of h-index, the two most productive institutions (Imperial College and UCL) occupied the two first places. According to MII, Oxford ranked first (1.403), followed by Edinburgh (1.296) and Cambridge (1.280).

**Table 2:** Number of publications (N), h-index, impact index () and modified impact index () for the top 10 European universities in life sciences and biomedicine according to Times Higher Education^1 ^(based on publications occurring during 2002–2006 from 36 medical fields).

University	Country	Rank according to THE^1^	N	h-index	Impact index	Modified impact index
Oxford	UK	2	5578	105	2.259	1.403

Edinburgh	UK	5	3318	77	2.088	1.296

Cambridge	UK	1	5605	96	2.061	1.280

Bristol	UK	9	3309	72	1.955	1.214

Imperial	UK	3	10624	118	1.906	1.184

Uppsala	Sweden	7	4073	77	1.906	1.183

Heidelberg	Germany	8	5785	86	1.821	1.131

Louis Pasteur Strasbourg I	France	10	1315	43	1.760	1.093

UCL	UK	4	12662	115	1.718	1.067

King's College	UK	6	8980	95	1.654	1.027

MII values > 1 indicate that an institution performs better in terms of its h-index that it would be expected, given the number of publications it produces.

^1 ^Times Higher Education – QS World University Rankings 2007 – Life Sciences and Biomedicine (rank among European Universities)

A higher heterogeneity was observed in the estimated MIIs for selected subfields such as e.g. in "Cardiac and Cardiovascular Systems" where MII was found to range within 0.842–1.720 (Table 3). Uppsala, Cambridge and Edinburgh ranked first according to MII in the subfields "Medicine, General and Internal", "Cardiac and Cardiovascular Systems" and "Infectious Diseases", respectively.

**Table 3:** Field-specific impact index and modified impact index for the top 10 European universities in life sciences and biomedicine according to Times Higher Education (7)

University	Country	N	h-index	Impact index	MII
**Medicine, General & Internal**					

Uppsala	Sweden	223	40	1.230	1.636

Cambridge	UK	428	50	1.010	1.344

Oxford	UK	601	60	0.974	1.296

Bristol	UK	434	47	0.941	1.252

Imperial	UK	1041	74	0.843	1.122

Edinburgh	UK	391	38	0.814	1.082

Heidelberg	Germany	204	24	0.781	1.039

UCL	UK	1385	76	0.721	0.959

Louis Pasteur Strasbourg I	France	98	12	0.626	0.833

King's College	UK	1063	54	0.607	0.808

**Cardiac and Cardiovascular Systems**					

Cambridge	UK	133	32	1.704	1.720

Edinburgh	UK	101	25	1.570	1.585

Louis Pasteur Strasbourg I	France	51	14	1.325	1.337

Oxford	UK	198	29	1.216	1.228

UCL	UK	452	46	1.176	1.187

Uppsala	Sweden	214	29	1.161	1.172

Bristol	UK	159	23	1.100	1.111

Heidelberg	Germany	323	31	0.970	0.979

King's College	UK	452	35	0.895	0.903

Imperial	UK	860	48	0.834	0.842

**Infectious Diseases**					

Edinburgh	UK	149	24	1.413	1.189

Oxford	UK	238	31	1.400	1.178

Bristol	UK	141	21	1.276	1.073

King's College	UK	212	26	1.254	1.055

Uppsala	Sweden	100	16	1.181	0.993

Cambridge	UK	145	19	1.136	0.956

Heidelberg	Germany	85	14	1.133	0.953

UCL	UK	709	42	1.023	0.861

Louis Pasteur Strasbourg I	France	49	9	0.994	0.837

Imperial	UK	739	40	0.951	0.801

## Discussion

The h-index is a valuable bibliometric indicator that combines information on both the quantity and the quality of the research output. Moreover, the findings of a recent paper indicate that it is better in predicting researchers' future scientific achievement than other indicators (total citation count, average number of citations per paper, total paper count) [9]. However, the h-index has various shortcomings, in particular when comparing individual scientists, discussed in detail by others [10-13]; it cannot differentiate between active and inactive scientists, it depends on the scientific age, it is affected by different discipline-dependent citation patterns etc. Numerous variants have been proposed that aim to overcome some of these disadvantages. For example, the m quotient allows to compare different lengths of scientific career [1], the g and h(2) indices give more weight to highly cited papers [14,15], the impact index h_m _provides an evaluation of the impact of the production [2] and the contemporary h-index [13] gives more weight to newer articles.

The proposed index deals with the fact that the inherent association of the h-index with the size of the research output may result in rewarding high production when evaluating institutions of disparate sizes. By definition, the h-index cannot exceed the number of publications. Thus, as noted by Glanzel [12] "it puts small but highly-cited paper sets at a disadvantage ('small is not beautiful')". An institution with a moderate-size production will not reach the h-index of a very large institution even if the quality of its publications are of similar or even better quality simply because its total production may be even less than *h*.

An application of the proposed modified impact index was presented using biomedical data. In biomedical research, the parameter *β *that characterises the dependence of h-index on the number of publications was approximately 0.4 and similar to that estimated in other disciplines (interdisciplinary, mechanics and materials science data [2], nonbiomedical research data [5] and chemical research data [16]). These estimates were based on publications ranging from a few hundreds to several thousands. When the number of publications ranges from a few papers up to approximately 500, as e.g. when evaluating the research output within specific subfields, the parameter *β *was higher than the overall estimate of 0.445. This was also noted by Molinari & Molinari [2] who have shown that the slope of the line describing the dependence of the h-index on the number of publications is higher when the number of evaluated papers is small. For example, in the field "Medicine, General & Internal" Uppsala had 223 papers with an h-index of 40, so using the appropriate field-specific values for the intercept α who have shown that the slope of the line describing the dependence of the h-index on the number of publications is higher when the number of evaluated papers is small.  In our biomedical data, the field-specific slopes ranged from 0.488 to 0.668. For example, in the field "Medicine, General & Internal" Uppsala had 223 papers with an h-index of 40, so using the appropriate field-specific values for the intercept *a *and slope *β *the corresponding MII was calculated to be .

The proposed index correlated with the share of government budget appropriations or outlays for research and development as % of GDP in 2004 (r = 0.229) whereas the corresponding correlation coefficient for the h-index was close to 0. Additionally, it was positively associated with the average number of citations/publication, the h-index and the number of highly cited papers. Furthermore, for a given *β *the MII provides the same ranking as the impact index proposed by Molinari and Molinari [2]. Actually, the estimates of *β *provided here can be used to calculate the impact index of institutions in biomedical research and within specific biomedical disciplines. Both indices have the advantage that they can be well estimated by using a representative subset of the publications rather than the total set of publications produced by an institution [2]. The advantage of MII over the impact index is its conceptual interpretation.

The estimates of *α *s and *β *s were based on data from European Medical Institutions. In order to assess whether these estimates can be used to calculate the MII for non-European institutions too, we performed a preliminary analysis to check whether the slope based on data from top-ranked US universities is similar to that obtained from the top-ranked European ones. We observed that these slopes were similar unless universities with number of publications outside the evaluated range were included (e.g. Harvard and Johns Hopkins). Thus, we advocate that the estimates provided here can be used to calculate the MII for non-European institutions, as long as their number of publications falls within the evaluated range (10^2^–10^4 ^papers for the 36 fields).

Bibliometric methods have been criticised due to technical and methodological problems generally encountered when they are employed to assess the research output of a university (17,18). Furthermore, the bibliometric indices currently used appear to be related to the size of research output and thus they probably tend to favour large institutions. The proposed index presents some clear advantages compared to existing bibliometric indices: it is not associated with the size of the publication output and thus can be used to compare institutions of disparate size, it has a conceptual interpretation (performance below or above the average) and can be computed by using a representative subset of the publications rather than the total set of publications produced by an institution. However, its computation requires estimates for the *α *s and *β *s and thus is not as straightforward as in the case of usual bibliometric indices. As mentioned before, the parameter *β *has a "universal" estimate of 0.4 independent of the discipline but dependent on the size of the publication set. As a result, the estimates for the α as a "universal" estimate of  0.4 independent of the discipline but dependent on the size of the publication set. As a result, the estimates for the *a *s and *β *s, as e.g. those provided here for biomedicine, can be applied to compute the MII of an institution as long as the number of its publications falls within the evaluated range (e.g. 10^2^–10^4 ^papers in our case). Thus, it would not be safe to use them for outliers, i.e. for institutions with productivity outside the evaluated range.

## Conclusion

In conclusion, there is a growing demand for transparent and valid evaluation of universities but any ranking is bound to give rise to controversy. The assessment of medical research performance, in particular, is a challenging task. Peer-review, the currently thought gold standard of research evaluation is usually not feasible for large-scale evaluations. For large-scale evaluative purposes, we advocate the use of a combination of bibliometric indices that will include an indicator not associated with the size of the research output. The proposed modified impact index is such an indicator that has a conceptual interpretation and with the data provided here can be computed for large as well as for small field-specific publication sets in biomedicine.

## Abbreviations

MII: Modified Impact Index

## Competing interests

The authors declare that they have no competing interests.

## Authors' contributions

VS oversaw the data collection and advised on the search strategy, analysed the data and co-wrote the first and subsequent drafts. AH conceived of the study, advised on the search strategy, oversaw data analysis and co-wrote the first and subsequent drafts. All authors read and approved the final manuscript.

## Pre-publication history

The pre-publication history for this paper can be accessed here:

http://www.biomedcentral.com/1471-2288/9/33/prepub
